# Effects of dietary supplementation of myristic acid on jejunal mucosa‐associated microbiota, mucosal immunity, and growth performance of nursery pigs

**DOI:** 10.1111/asj.70027

**Published:** 2025-01-08

**Authors:** Hyunjun Choi, Gabriel Cipriano Rocha, Sung Woo Kim

**Affiliations:** ^1^ Department of Animal Science North Carolina State University Raleigh NC USA

**Keywords:** antibiotic, growth performance, mucosal immunity, myristic acid, nursery pig

## Abstract

The objective of this study was to investigate the effects of myristic acid on jejunal mucosal microbiota, mucosal immunity, and growth performance of nursery pigs. Thirty‐six pigs (6.6 ± 0.4 kg of body weight) were assigned to three treatments (*n* = 12) for 35 d in three phases: (NC) basal diet; (PC) NC + bacitracin; and (MA) NC + myristic acid compound. Pigs were euthanized to collect jejunal mucosa, jejunal tissues, and ileal digesta. The PC increased (*p* < 0.05) the relative abundance (RA) of *Lactobacillus* spp., and *Bifidobacterium boum* than the NC group. The MA increased (*p* < 0.05) RA of *Bifidobacterium dentium* and *Megasphaera* spp. than the NC group. The PC tended to decrease IL‐8 (*p* = 0.053) and protein carbonyl (*p* = 0.075) whereas IgG (*p* = 0.051) and IL‐8 (*p* = 0.090) in jejunal mucosa were decreased by the MA. The PC increased (*p* < 0.05) the villus height to crypt depth ratio than the NC group. Both bacitracin and myristic acid improved the intestinal health and growth performance of nursery pigs. Effects of bacitracin were rather immediate whereas the effects of myristic acid were obtained after a 3‐week feeding.

## 1 INTRODUCTION

Weaning is a critical event for the intestinal health and growth of pigs (Moeser et al., 2017; Pluske, 2016). Newly weaned pigs are exposed to various challenges during the post‐weaning period, including environmental, immunological, behavioral, and nutritional factors (Duarte & Kim, 2022a; Jang et al., 2021; Main et al., 2004). Weaning stress can have negative impacts on the intestinal microbiota, intestinal health, and growth performance of pigs (Kim & Duarte, 2021). Antibiotics have been extensively used in swine feeds to mitigate the negative impacts of weaning on the intestinal health and growth performance of pigs (Cromwell, 2002). However, due to the concerns about microbial resistance of bacteria, the use of antibiotics in feed as growth promoters has been phased out (Casewell et al., 2003; Tiseo et al., 2020).

Myristic acid is a saturated fatty acid with 14 carbon atoms and has been shown to have antimicrobial effects, particularly on Gram‐positive pathogenic bacteria (Qi et al., 2023). Myristic acid can be attached to the cell wall of opportunistic pathogenic bacteria and change bacterial cell membrane permeability, thereby showing antimicrobial activity, consequently modulating the microbial diversity and microbial composition in chickens (Chen et al., 2019; Liu & Huang, 2012). Myristic acid showed high bactericidal activity, reducing *Clostrium perfringens* (Qi et al., 2023) and *Staphylococcus aureus* (Park et al., 2022), which can have potentially negative impacts on the intestinal health and growth of pigs (Deplancke et al., 2002; Reznikov et al., 2018). The mucosa‐associated microbiota interacts directly with the mucus layer and intestinal cells, whereas the luminal microbiota could do that indirectly through their metabolites in the small intestine of pigs (Arpaia et al., 2013; Duarte & Kim, 2022c). Myristic acid is also known to be a ligand for G‐protein‐coupled receptor (GPR)84 located in the small intestine of pigs (Wang et al., 2006), which positively modulates immune responses (Zhang, Chen, et al., 2022). Myristic acid is mainly obtained from coconut oil and black soldier fly larvae oil, which contains 16% to 21% and 6% to 25% myristic acid, respectively (Dubois et al., 2007; Kim et al., 2021). Previous studies have reported that replacing corn oil with coconut oil or black soldier fly larvae oil improved growth performance in chickens (Kim et al., 2020), and replacing corn oil with black soldier fly larvae oil improved growth performance in pigs (van Heugten et al., 2022), potentially due to the antimicrobial properties of these oils (Chen et al., 2022; Dayrit, 2015). However, coconut oil and black soldier fly larvae oil also contain 45 to 52% and 27 to 41% lauric acid, respectively (Kim et al., 2021; Nitbani et al., 2022), which complicates understanding the specific effects of myristic acid on the intestinal microbiota, intestinal health, and growth of nursery pigs. Thus, the dietary supplementation with myristic acid could have antimicrobial properties in the jejunum of pigs, positively modulating the composition of jejunal mucosa‐associated microbiota and consequently promoting intestinal health and growth of nursery pigs. Based on the previous findings, it is hypothesized that myristic acid, as an antibiotic alternative, would have antimicrobial properties in the jejunum by positively modulating jejunal mucosa‐associated microbiota and thus improving intestinal health and growth performance of nursery pigs. To test the hypothesis, the objective of this study was to investigate the effects of dietary myristic acid on jejunal mucosa‐associated microbiota, intestinal health, and growth performance of nursery pigs.

## 2 MATERIALS AND METHODS

The experimental procedure was reviewed and approved by the North Carolina State University Animal Care and Use Committee (Raleigh, NC, USA).

### 2.1 Animals, experimental design, and experimental diets

A total of 36 newly weaned pigs (18 barrows and 18 gilts) at 21 d of age with initial body weight (BW) at 6.6 ± 0.4 kg were assigned to 3 dietary treatments in a randomized complete block design with initial BW and sex as blocks. Each treatment had 12 replicates (6 pens with barrows and 6 pens with gilts). Pigs were individually housed in pens (1.50 m × 0.74 m) and had free access to feeds and water throughout the experimental period. There were 3 treatments 1) NC: basal diet; 2) PC: NC + 0.25% bacitracin methylene disalicylate (antibiotic; bacitracin: 0.03%) in all phases; and 3) MA: NC + myristic acid compound at 0.20% in phases 1 and 2 and 0.12% in phase 3 (Table 1). Myristic acid compound contains 30% to 45% myristic acid and other compounds are 40% to 50% zeolite power and 10% to 15% silicon dioxide. Each feed additive was supplemented to the NC at the expense of corn to make the experimental diets. The experimental diets were formulated to meet or exceed the nutrient requirement estimates of pigs (NRC, 2012) in three phases: phase 1 (d 0 to 10), phase 2 (d 10 to 20), and phase 3 (d 20 to 35). The nutrient composition in feedstuffs used in the experimental diets, including metabolizable energy, standardized ileal digestible amino acids, total calcium (Ca), and standardized total tract digestible phosphorus (P), were taken from NRC (2012). Experimental diets did not contain zinc and copper at pharmacological levels and were in a mash form. Titanium dioxide (TiO_2_) was supplemented at 0.40% to the phase three diets from d 28 to 35 as an indigestible marker to determine the apparent ileal digestibility (AID) of energy and nutrients.

**TABLE 1:** Composition of basal diets (as‐fed basis).

Item	Phase 1	Phase 2	Phase 3^a^
Feedstuff, %			
Corn, yellow dent	31.54	45.80	67.95
Soybean meal, 48% crude protein	19.00	23.00	26.00
Whey permeate	20.00	14.00	‐
Poultry meal	7.00	3.00	‐
Bakery meal	10.00	5.00	‐
Fish meal	4.00	2.00	‐
Blood plasma	4.00	2.00	‐
Poultry fat	2.00	2.00	2.00
L‐Lys HCl	0.50	0.50	0.54
L‐Met	0.24	0.20	0.18
L‐Thr	0.16	0.16	0.18
L‐Trp	0.02	0.02	0.02
L‐Val	‐	0.04	0.08
Dicalcium phosphate	0.02	0.64	1.30
Limestone	0.62	0.74	0.85
Trace mineral premix^b^	0.15	0.15	0.15
Vitamin premix^c^	0.03	0.03	0.03
Sodium chloride	0.22	0.22	0.22
Feed additive mixture^d^ (bacitracin or myristic acid)	0.50	0.50	0.50
Calculated composition			
Dry matter, %	91.02	90.32	89.38
ME, kcal/kg	3,484	3,425	3,388
Crude protein, %	24.18	21.20	18.90
SID Lys, %	1.51	1.36	1.24
SID Met, %	0.84	0.75	0.69
SID Thr, %	0.88	0.80	0.74
SID Trp, %	0.26	0.24	0.21
SID Val, %	0.97	0.87	0.80
Total calcium, %	0.86	0.80	0.72
STTD phosphorus, %	0.46	0.41	0.34
Total phosphorus, %	0.68	0.64	0.60
Analyzed composition			
Dry matter, %	89.77	88.67	87.19
Gross energy, kcal/kg	‐	‐	3,911
Crude protein, %	22.31	20.90	17.00
Neutral detergent fiber, %	9.27	9.97	6.71
Acid detergent fiber, %	2.81	3.34	2.85
Ether extract, %	5.89	5.37	4.66

Abbreviations: ME, metabolizable energy; SID, standardized ileal digestible; STTD, standardized total tract digestible.

^a^ Titanium dioxide was supplemented at 0.40% to the experimental diets as an indigestible marker during d 28 to 35.

^b^ The trace mineral premix provided per kilogram of complete diet: 33 mg of Mn as manganous oxide, 110 mg of Fe as ferrous sulfate, 110 mg of Zn as zinc sulfate, 16.5 mg of Cu as copper sulfate, 0.30 mg of I as ethylenediamine dihydroiodide, and 0.30 mg of Se as sodium selenite.

^c^ The vitamin premix provided per kilogram of complete diet: 6,614 IU of vitamin A as vitamin A acetate, 992 IU of vitamin D_3_, 19.8 IU of vitamin E, 2.64 mg of vitamin K as menadione sodium bisulfate, 0.03 mg of vitamin B_12_, 4.63 mg of riboflavin, 18.52 mg of D‐pantothenic acid as calcium panthonate, 24.96 mg of niacin, and 0.07 mg of biotin.

^d^ There were three dietary treatments: 1) NC: basal diet, 2) PC: NC + 0.25% bacitracin methylene disalicylate (antibiotic; bacitracin: 0.03%) in phases 1 to 3, and 3) MA: NC + myristic acid compound at 0.20% in phases 1 and 2 and 0.12% in phase 3. Antibiotic and myristic acid were supplemented at the expense of corn in the basal diets.

### 2.2 Sample and data collection

The BW of the pigs and feed disappearance were measured on d 0, 10, 20, and 35 to determine average daily gain (ADG), average daily feed intake (ADFI), and gain‐to‐feed ratio (G:F) for the growth performance. Fecal score of each pen was recorded based on a 1 to 5 scale (1: firm and 5: watery) by visual observation of fresh feces from day 3 at 2‐d intervals (Choi et al., 2024). At d 35, all pigs were euthanized by the penetration of a captive bolt followed by exsanguination. After euthanasia, jejunal mucosa, jejunal tissues, and ileal digesta were collected. Jejunal tissues were obtained from 3 to 4 m after the pyloric valve of the stomach of pigs. The mid‐jejunal tissues (20 cm) also were flushed with 0.9% saline solution to remove jejunal digesta and the flushed jejunal tissues were collected. The first 15 cm was used to collect jejunal mucosa by scraping the mucosa layer in the jejunum using a glass microscope slide and the remaining 5 cm was fixed in 10% buffered formaldehyde to be used for Ki‐67^+^ staining and histological evaluation. Jejunal mucosa samples were collected for tumor necrosis factor‐alpha (TNF‐α), interleukin‐8 (IL‐8), immunoglobulin A (IgA), immunoglobulin G (IgG), and high mobility group protein B1 (HMGB‐1) as indicators of immune response status and protein carbonyl and malondialdehyde (MDA) as indicators of oxidative damage products. In addition, the jejunal mucosal samples were transferred to the freezer at −80°C for further DNA extraction and quantification of immune and oxidative damage products. Ileal digesta was collected in a 50 ml container and stored at −20°C for further process and analysis for AID of energy and nutrients.

### 2.3 Diversity and relative abundance of the mucosa‐associated microbiota in the jejunum

The jejunal mucosa samples were sent to Zymo Research Corporation (Irvine, CA, USA) to determine alpha diversity and relative abundance (RA) of mucosa‐associated microbiota in the jejunum. Jejunal mucosa samples were used for DNA extraction for 16S rRNA sequencing using the ZymoBIOMICS‐96 MagBead DNA kit (Zymo Research). The extracted DNA samples were prepared for targeted sequencing with the Quick‐16S Primer Set V3‐V4 (Zymo Research) and NGS library Preparation Kit for microbial analysis. These primers are custom‐designed by Zymo Research to provide the best coverage of the 16S gene. The final PCR products were quantified with qPCR fluorescence readings and pooled together based on equal molarity. The final pooled library was cleaned up with the Select‐a‐Size DNA Clean & Concentrator (Zymo Research), then quantified with TapeStation (Agilent Technologies, Santa Clara, CA, USA) and Qubit (Thermo Fisher Scientific, Waltham, WA, USA). For sequencing, the final library was sequenced on Illumina NextSeq 2000 (Illumina, San Diego, CA, USA) with a p1 (cat 20,075,294) reagent kit (600 cycles). The sequencing was performed with 30% PhiX spike‐in using the Phix Control kit V3. Unique amplicon sequences were inferred from raw reads using the DADA2 pipeline (Callahan et al., 2016). Chimeric sequences were also removed with the DADA2 pipeline. The depth of sequencing coverage was > 20,000 × sample. Taxonomy was assigned with the Zymo Research Database, a 16S databased that is internally designed and curated, as a reference. Alpha diversity (Chao 1, Shannon, and Simpson index) and beta diversity (Bray‐Curtis distance) were evaluated with MicrobiomeAnalyst (QC, CA). The ASV data were transformed to RA for further statistical analysis, and the ASV data with less than 0.5% abundance within each level were combined as “others”.

### 2.4 Immune response and oxidative damage products in the jejunum

One gram of jejunal mucosa sample was weighed and ground using a homogenizer (Tissuemiser, Thermo Fisher Scientific Inc., IL, USA) on ice in 2 ml phosphate‐buffered saline for 30 s. The homogenate was centrifuged at 14,000×*g* for 30 min at 4°C to obtain supernatant, which was used to determine the contents of total protein, IgA, IgG, TNF‐α, IL‐8, protein carbonyl, MDA, and HMGB‐1. The supernatant was pipetted off and kept at −80°C. The content of total protein of mucosa was determined using the kit Pierce BCA Protein Assay (23,225#, Thermo Fisher Scientific Inc.) to calculate the contents of IgA, IgG, TNF‐α, IL‐8, protein carbonyl, MDA, and HMGB‐1 per milligram of protein in the jejunal mucosa sample. The content of IgA and IgG was analyzed using an ELISA kit for pig IgA (E101–102, Bethyl Laboratories, Inc, Montgomery, TX, USA) and pig IgG (E101–104, Bethyl Laboratories, Inc.), respectively. The mucosal samples were diluted to 1:1,000 and 1:1,600 with phosphate‐buffered saline to analyze IgA and IgG contents, respectively. The contents of MDA and protein carbonyl were measured by commercial assay kits (Cell Biolabs, Inc., San Diego, CA, USA) following the protocols of the manufacturer. The contents of TNF‐α and IL‐8 in jejunal mucosa were measured by ELISA kits (R&D Systems, Minneapolis, MN, USA) following Jang and Kim (2019). The content of HMGB‐1 was measured by an ELISA kit (Mybiosource, Inc., San Diego, CA, USA).

### 2.5 Intestinal morphology and crypt cell proliferation in the jejunum

After 48 h in 10% buffered formaldehyde solution, two sections of the jejunum per pig were transversely cut, placed into a cassette in 70% ethanol, and sent to the University of North Carolina Histology Laboratory (UNC School of Medicine, Chapel Hill, NC, USA) for dehydration, embedment, and Ki‐67^+^ immunohistochemistry staining for morphological evaluation and to evaluate cell proliferation in the crypt. Pictures of villus and crypts were taken at 40 × magnification and were measured villus height (VH) and crypt depth (CD) using a camera Infinity 2–2 digital CCD attached to a microscope Olympus CX31 (Lumenera Corporation, Ottawa, Canada) for intestinal morphology of pigs. The ratio of VH to CD (VH:CD) was also calculated. Pictures of crypts in 100 × magnification were taken for Ki‐67^+^ cell measurement. The ImageJS software was used for calculating the percentage of dyed Ki‐67^+^ cells in the total cells in the crypt. The percentage of Ki‐67^+^ cells was used as an indicator of enterocyte proliferation in the crypt. All analyses of the intestinal morphology were executed by the same person, and the average 15 measurements of each sample were calculated and reported as one number per sample.

### 2.6 Chemical analysis

Frozen ileal digesta were dried in a freeze drier. Experimental diets and dried ileal digesta were finely ground and analyzed for dry matter (DM; method 930.15) and ether extract (method 920.39) as described in AOAC (2005). Nitrogen (N) contents in the diets and ileal digesta were measured using a TrueSpec N Nitrogen Determinator (LECO Corp., St. Joseph, MI, USA) to calculate crude protein (CP, 6.25 × N). Experimental diets and ileal digesta were analyzed for gross energy (GE) using a bomb calorimetry (Parr 1,261, Parr Instrument Co., Moline, IL, USA). Experimental diets were analyzed for neutral detergent fiber (method 2002.04) and acid detergent fiber (method 973.18) as described in AOAC (2005), using an ANKOM 200 Fiber Analyzer (Ankom Technology Corp., Macedon, NY, USA).

### 2.7 Apparent ileal digestibility of energy and nutrients

The TiO_2_ contents of the diet and ileal digesta were determined by the procedure described in Chen et al. (2017). Briefly, the diet and ileal digesta samples were digested in Kjeldahl digestion tubes with a catalyst and 13 ml of concentrated sulfuric acid at 420°C for 2 h. After cooling for 30 min, 10 ml of hydrogen peroxide was added to each tube, and the total liquid volume was adjusted to 100 ml with distilled water. The liquid was transferred to a microplate to determine TiO_2_ at 410 nm using a microplate spectrophotometer. The AID of GE was calculated according to the following equation (Choi et al., 2024):

AID of GE, % = [1 − (TiO_2diet_/TiO_2digesta_) × (GE_digesta_/GE_diet_)] × 100 where TiO_2diet_ and TiO_2digesta_ are the TiO_2_ contents in the diet and ileal digesta, respectively (g/kg; DM basis); and GE_digesta_ and GE_diet_ are the energy contents of the ileal digesta and diet, respectively (kcal/kg; DM basis). The AID values of nutrients (DM, CP, and EE) were also calculated using the same equation. Nutrient contents (DM basis) were expressed as gram per kilogram.

### 2.8 Statistical analyses

Experimental data were analyzed using the MIXED procedure (SAS Inst. Inc., Cary, NC, USA). The statistical model included dietary treatment as a fixed effect and initial BW and sex as random effects. A power test was conducted to determine the number of replications needed for the study. To determine the statistical significance of the expected mean difference of 10 to 11% at *p* < 0.05, using coefficient of variation at 7.5% based on previous studies conducted using pigs with similar genetic background and research environment (Deng et al., 2023; Duarte et al., 2019) and the power of test (1 – beta) at 95% the power analysis indicated an 80%, the minimum number of replications for each treatment was 12 (Aron & Hays, 2004). An observation of a pig fed the NC diet was removed from the data set for the final analysis as the observation deviated by more than 1.5 times the interquartile ranges from the treatment median value of growth performance, showing feed refusal. The least squares mean of each treatment was calculated. Differences between least squares means were determined by the preplanned contrasts, which were made between NC vs. PC and NC vs. MA. Spearman correlation coefficients among the mucosa‐associated microbiota, immune response, oxidative damage products, intestinal morphology, and growth performance were determined by the CORR Spearman procedure of SAS. The analysis of similarities (ANOSIM) was performed to evaluate the beta diversity of mucosa‐associated microbiota. The data were visualized using principal coordinate analysis (PCoA) based on the Bray‐Curtis distance. The experimental unit was a pen. The statistical significance and tendency were declared at *p* < 0.05 and 0.05 ≤ *p* < 0.10, respectively.

## 3 RESULTS

### 3.1 Diversity and relative abundance of the mucosa‐associated microbiota in the jejunum

**FIGURE 1:**
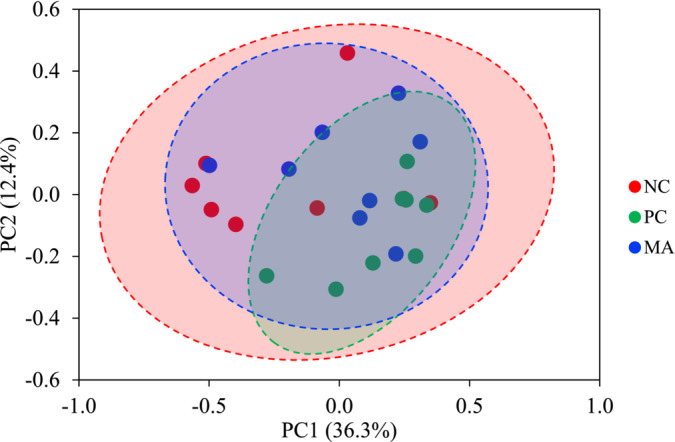
Principal component analysis (PCoA) plot in the jejunal mucosa‐associated microbiota at the species level in nursery pigs fed diets with antibiotic and myristic acid. The X‐axis and Y‐axis represent the principal component axes, with the percentages indicating the proportion of variation explained by each component. Points of different colors correspond to samples from different treatments (NC, PC, and MA), and the closer two points are, the more similar their species composition. The *p*‐value for Bray‐curtis between NC and PC was less than 0.05, and NC and MA was less than 0.05.

The PC increased (*p* < 0.05) Shannon and Simpson indexes and the MA increased (*p* < 0.05) Chao1, Shannon, and Simpson indexes compared with the NC group (Table 2). The microbial community was visualized using PCoA based on Bray‐Curtis distance, which confirmed that the PC changed (*p* < 0.05) the composition of mucosa‐associated microbiota in the jejunum of nursery pigs compared to the NC group (Figure 1). The beta diversity of the jejunal mucosa‐associated microbiota in the MA was different (*p* < 0.05) from that of the NC group. The PC increased (*p* < 0.05) RA of Firmicutes and decreased (*p* < 0.05) that of Proteobacteria compared with the NC (Table 3). The MA tended to decrease the RA of Proteobacteria (*p* = 0.059) and Actinobacteria (*p* = 0.051) compared with the NC group. The PC increased (*p* < 0.05) Lactobacillaceae, tended to increase (*p* = 0.075) Veillonellaceae, and tended to decrease (*p* = 0.096) Xanthomonadaceae compared with the NC group. The MA increased (*p* < 0.05) Veillonellaceae, Lachnospiraceae, Coriobacteriaceae, and Ruminococcaceae compared with the NC group. Additionally, The MA tended to increase Bifidobacteriaceae (*p* = 0.072) and Streptococcaceae (*p* = 0.076) compared with the NC group. The PC increased (*p* < 0.05) *Lactobacillus* and *Miitsuokella*, and tended to decrease (*p* = 0.099) *Stenotrophomonas* compared with the NC group (Table 4). The MA increased (*p* < 0.05) *Megasphaera* and *Stenotrophomonas*, and tended to increase *Bifidobacterium* (*p* = 0.072), *Streptococcus* (*p* = 0.076), and *Olsenella* (*p* = 0.089) compared with the NC group. The PC increased (*p* < 0.05) *Lactobacillus* spp., *Bifidobacterium boum*, and *Mitsuokella multacida* compared with the NC group (Table 5). The MA increased (*p* < 0.05) *Bifidobacterium dentium* and *Megasphaera* spp. compared with the NC group.

**TABLE 2:** Alpha diversity of jejunal mucosa‐associated microbiota at the species level in nursery pigs fed diets with antibiotic and myristic acid.

Item	Treatment^a^			SEM	*p*‐value	
	NC	PC	MA		NC vs. PC	NC vs. MA
Chao 1	37.3	44.6	59.2	5.1	0.264	0.003
Shannon	1.88	2.33	2.58	0.15	0.045	0.004
Simpson	0.71	0.85	0.85	0.04	0.013	0.014

Abbreviation: SEM, standard error of the mean.

^a^ NC: basal diet (no supplementation); PC: NC + bacitracin methylene disalicylate (antibiotic; bacitracin: 0.03% feed); MA: NC + myristic acid. Experimental unit was a pig. Each least square mean represent 12 observations except for NC that represent 11 observations.

**TABLE 3:** Relative abundance of jejunal mucosa‐associated microbiota at the phylum and family levels in nursery pigs fed diets with antibiotic and myristic acid.

Item	Treatment^a^			SEM	*p*‐value	
	NC	PC	MA		NC vs. PC	NC vs. MA
Phylum						
Firmicutes	38.90	65.88	54.71	9.27	0.030	0.183
Proteobacteria	45.63	13.88	18.16	9.65	0.031	0.059
Actinobacteria	7.62	17.34	20.37	4.32	0.128	0.051
Bacteroidetes	2.26	0.12	3.44	1.39	0.227	0.500
Chlamydiae	1.49	0.28	<0.01	0.80	0.237	0.139
Others	3.90	2.21	3.15	1.31	0.287	0.631
Family						
Lactobacillaceae	22.20	55.13	34.27	7.44	0.006	0.266
Helicobacteraceae	33.43	12.78	16.00	9.32	0.103	0.163
Bifidobacteriaceae	6.86	16.11	18.20	4.21	0.137	0.072
Staphylococcaceae	10.44	0.49	0.61	6.00	0.256	0.261
Veillonellaceae	1.67	4.16	5.15	0.93	0.075	0.016
Streptococcaceae	1.54	1.68	3.64	0.98	0.902	0.076
Pseudomonadaceae	6.82	0.03	0.02	3.82	0.224	0.223
Lachnospiraceae	0.33	1.33	3.55	0.58	0.219	0.001
Erysipelotrichaceae	1.52	0.97	1.51	0.49	0.434	0.987
Prevotellaceae	1.38	0.05	2.68	1.05	0.293	0.302
Coriobacteriaceae	0.56	1.02	1.46	0.28	0.243	0.027
Ruminococcaceae	0.46	0.19	2.11	0.57	0.633	0.009
Peptostreptococcaceae	0.26	0.55	1.49	0.81	0.804	0.296
Xanthomonadaceae	1.46	0.32	<0.01	0.60	0.096	0.034
Chlamydiaceae	1.49	0.28	<0.01	0.80	0.237	0.139
Burkholderiaceae	0.76	0.21	0.81	0.33	0.160	0.908
Others	9.27	5.17	9.02	2.97	0.331	0.951

Abbreviation: SEM, standard error of the mean.

^a^ NC: basal diet (no supplementation); PC: NC + bacitracin methylene disalicylate (antibiotic; bacitracin: 0.03% feed); MA: NC + myristic acid. Experimental unit was a pig. Each least square mean represent 12 observations except for NC that represent 11 observations.

**TABLE 4:** Relative abundance of jejunal mucosa‐associated microbiota at the genus level in nursery pigs fed diets with antibiotic and myristic acid.

Item	Treatment^a^			SEM	*p*‐value	
	NC	PC	MA		NC vs. PC	NC vs. MA
*Lactobacillus*	22.20	55.13	34.27	7.44	0.006	0.266
*Helicobacter*	33.43	12.78	16.00	9.32	0.103	0.163
*Bifidobacterium*	6.86	16.11	18.20	4.21	0.137	0.072
*Staphylococcus*	10.44	0.49	0.61	6.00	0.255	0.261
*Streptococcus*	1.54	1.68	3.64	0.98	0.902	0.076
*Pseudomonas*	6.82	0.03	0.02	3.82	0.224	0.223
*Megasphaera*	0.72	1.79	2.42	0.59	0.187	0.041
*Mitsuokella*	0.30	1.83	0.85	0.39	0.003	0.234
*Olsenella*	0.40	0.84	1.00	0.26	0.204	0.089
*Chlamydia*	1.49	0.28	<0.01	0.80	0.237	0.139
*Stenotrophomonas*	1.44	0.32	<0.01	0.60	0.099	0.034
*Prevotella*	0.34	<0.01	1.27	0.53	0.597	0.159
Others	14.42	9.20	22.17	5.09	0.449	0.264

Abbreviation: SEM, standard error of the mean.

^a^ NC: basal diet (no supplementation); PC: NC + bacitracin methylene disalicylate (antibiotic; bacitracin: 0.03% feed); MA: NC + myristic acid. Experimental unit was a pig. Each least square mean represent 12 observations except for NC that represent 11 observations.

**TABLE 5:** Relative abundance of jejunal mucosa‐associated microbiota at the species level in nursery pigs fed diets with antibiotic and myristic acid.

Item	Treatment^a^			SEM	*p*‐value	
	NC	PC	MA		NC vs. PC	NC vs. MA
*Helicobacter rappini*	20.43	9.97	12.68	8.01	0.306	0.443
*Lactobacillus mucosae*	6.44	10.02	7.21	2.85	0.386	0.851
*Lactobacillus* spp.	3.51	11.66	3.94	2.61	0.040	0.909
*Lactobacillus delbrueckii*	1.99	2.52	7.13	3.15	0.873	0.128
*Bifidobacterium boum*	3.36	11.43	3.15	2.21	0.018	0.947
*Bifidobacterium thermacidophilum*	2.20	4.67	6.64	2.21	0.439	0.172
*Lactobacillus equicursoris*	1.48	5.30	2.94	2.26	0.148	0.568
*Helicobacter canadensis‐equorum*	10.28	<0.01	0.27	6.03	0.210	0.227
*Bifidobacterium dentium*	1.19	0.16	8.30	2.16	0.710	0.017
*Helicobacter equorum*	2.15	3.12	2.81	2.42	0.777	0.846
*Staphylococcus saprophyticus‐xylosus*	6.87	0.33	0.32	3.94	0.255	0.254
*Megasphaera* spp.	0.72	1.77	2.37	0.59	0.189	0.045
*Pseudomonas baetica‐fluorescens*	4.04	0.01	0.00	2.33	0.237	0.236
*S. saprophyticus*	3.56	0.14	0.17	2.06	0.255	0.259
*Lactobacillus salivarius*	0.45	1.60	1.47	0.51	0.131	0.176
*Chlamydia muridarum*	1.49	0.28	<0.01	0.80	0.237	0.139
*Ralstonia pickettii*	0.74	0.21	0.81	0.33	0.169	0.850
*Stenotrophomonas maltophilia*	1.30	0.30	<0.01	0.54	0.103	0.034
*Mitsuokella multacida*	0.04	1.00	0.45	0.29	0.006	0.198
Others	25.31	24.01	35.32	7.40	0.880	0.252

Abbreviation: SEM, standard error of the mean.

^a^ NC: basal diet (no supplementation); PC: NC + bacitracin methylene disalicylate (antibiotic; bacitracin: 0.03% feed); MA: NC + myristic acid; experimental unit was a pig; each least square mean represent 12 observations except for NC that represent 11 observations.

### 3.2 Immune response and oxidative damage products in the jejunum

The PC tended to decrease the IL‐8 (*p* = 0.053) and protein carbonyl (*p* = 0.075) in jejunal mucosa compared with the NC group (Table 6). The MA tended to decrease the IgG (*p* = 0.051) and IL‐8 (*p* = 0.090) in the jejunal mucosa compared with that of the NC group. However, IgA, TNF‐a, MDA, and HGMB‐1 were not affected by the PC and MA.

**TABLE 6:** Immune responses and oxidative damage products in the jejunal mucosa of nursery pigs fed diets with antibiotic and myristic acid.

Item	Treatment^a^			SEM	*p*‐value	
	NC	PC	MA		NC vs. PC	NC vs. MA
Jejunal mucosa, /mg of protein						
IgA, μg	5.73	5.64	6.01	0.92	0.923	0.769
IgG, μg	2.44	1.94	1.67	0.60	0.192	0.051
IL‐8, ng	0.53	0.37	0.39	0.10	0.053	0.090
TNF‐α, pg	0.81	0.59	1.07	0.25	0.284	0.200
MDA, nmol	0.44	0.37	0.36	0.06	0.349	0.234
Protein carbonyl, nmol	2.65	1.66	2.10	0.93	0.075	0.323
HGMB‐1, ng	19.3	19.6	18.9	1.8	0.911	0.844

Abbreviations: SEM, standard error of the mean; IgA, immunoglobulin A; IgG, immunoglobulin G; IL‐8, interleukin 8; TNF‐α, tumor necrosis factor alpha; MDA, malondialdehyde; HGMB‐1, high mobility group protein B1.

^a^ NC: basal diet (no supplementation); PC: NC + bacitracin methylene disalicylate (antibiotic; bacitracin: 0.03% feed); MA: NC + myristic acid; experimental unit was a pig; each least square mean represent 12 observations except for NC that represent 11 observations.

### 3.3 Intestinal morphology and crypt cell proliferation in the jejunum

The PC increased (*p* < 0.05) the VH:CD compared with that of the NC group (Table 7). However, VH, CD, and Ki‐67^+^ were not affected by the PC and MA.

**TABLE 7:** Intestinal morphology and crypt cell proliferation in the jejunum of nursery pigs fed diets with antibiotic and myristic acid.

Item	Treatment^a^			SEM	*p*‐value	
	NC	PC	MA		NC vs. PC	NC vs. MA
Jejunum						
Villus height, μm	483	533	505	48	0.115	0.489
Crypt depth, μm	186	181	191	9	0.681	0.732
VH:CD	2.61	3.02	2.70	0.31	0.036	0.642
Ki‐67^+b^, %	28.3	28.2	28.3	0.9	0.929	0.996

Abbreviations: SEM, standard error of the mean; VH:CD: villus height to crypt depth ratio.

^a^ NC: basal diet (no supplementation); PC: NC + bacitracin methylene disalicylate (antibiotic; bacitracin: 0.03% feed); MA: NC + myristic acid; experimental unit was a pig; each least square mean represent 12 observations except for NC that represent 11 observations.

^b^ Ratio of Ki‐67 positive cell to total cell in the crypt, which represents crypt cell proliferation.

### 3.4 Apparent ileal digestibility of energy and nutrients

The AID of GE and nutrients (DM, CP, and EE) were not affected by the PC and MA (Table 8).

**TABLE 8:** Apparent ileal digestibility of energy and nutrients (%) in nursery pigs fed diets with antibiotic and myristic acid (DM basis).

Item	Treatment^a^			SEM	*p‐*value	
	NC	PC	MA		NC vs. PC	NC vs. MA
Dry matter	60.8	63.9	62.2	3.5	0.536	0.763
Gross energy	64.5	66.4	64.8	3.5	0.704	0.949
Crude protein	70.0	68.4	67.1	9.7	0.815	0.663
Ether extract	60.2	66.0	65.6	6.0	0.325	0.341

Abbreviation: SEM, standard error of the mean.

^a^ NC: basal diet (no supplementation); PC: NC + bacitracin methylene disalicylate (antibiotic; bacitracin: 0.03% feed); MA: NC + myristic acid; experimental unit was a pig; each least square mean represent 12 observations except for NC that represent 11 observations.

### 3.5 Growth performance and fecal score

The PC tended to decrease (*p* = 0.056) fecal score in phase 2 compared with the NC group (Table 9). Compared with the NC group, the PC improved (*p* < 0.05) ADG in phase 1 and ADFI in phase 2, whereas the MA improved (*p* < 0.05) ADG and ADFI in phase 3 (Table 10). The MA tended to improve ADG (*p* = 0.072) and ADFI (*p* = 0.053) compared with the NC group in the entire period. The PC improved (*p* < 0.05) G:F in phase 1 compared with the NC group. However, G:F in the entire period was not affected by the PC and MA.

**TABLE 9:** Fecal score of nursery pigs fed diets with antibiotic and myristic acid.

Item	Treatment^a^			SEM	*p‐*value	
	NC	PC	MA		NC vs. PC	NC vs. MA
Phase 1 (d 0 to 10)	3.91	3.90	3.99	0.15	0.965	0.601
Phase 2 (d 10 to 20)	3.17	3.03	3.06	0.05	0.056	0.147
Phase 3 (d 20 to 35)	3.04	3.02	3.02	0.03	0.572	0.572
Overall (d 0 to 35)	3.31	3.25	3.26	0.05	0.302	0.449

Abbreviation: SEM, standard error of the mean.

^a^ NC: basal diet (no supplementation); PC: NC + bacitracin methylene disalicylate (antibiotic; bacitracin: 0.03% feed); MA: NC + myristic acid; experimental unit was a pig; each least square mean represent 12 observations except for NC that represent 11 observations.

**TABLE 10:** Growth performance of nursery pigs fed diets with antibiotic and myristic acid.

Item	Treatment^a^			SEM	*p‐*value	
	NC	PC	MA		NC vs. PC	NC vs. MA
Body weight, kg						
Day 0	6.7	6.6	6.6	0.3	0.734	0.601
Day 10	7.2	7.6	7.3	0.3	0.075	0.636
Day 20	11.2	12.3	11.7	0.4	0.052	0.426
Day 35	20.0	21.3	21.7	0.7	0.190	0.091
Average daily gain, g/d						
Phase 1 (d 0 to 10)	49	101	68	18	0.045	0.469
Phase 2 (d 10 to 20)	405	469	439	30	0.132	0.417
Phase 3 (d 20 to 35)	585	599	668	25	0.692	0.023
Overall (d 0 to 35)	380	419	431	20	0.162	0.072
Average daily feed intake, g/d						
Phase 1 (d 0 to 10)	149	180	158	15	0.133	0.647
Phase 2 (d 10 to 20)	477	578	530	31	0.024	0.223
Phase 3 (d 20 to 35)	849	908	996	46	0.364	0.028
Overall (d 0 to 35)	543	606	623	29	0.126	0.053
Gain to feed ratio						
Phase 1 (d 0 to 10)	0.23	0.53	0.39	0.10	0.041	0.255
Phase 2 (d 10 to 20)	0.84	0.81	0.82	0.03	0.449	0.622
Phase 3 (d 20 to 35)	0.69	0.66	0.68	0.02	0.144	0.464
Overall (d 0 to 35)	0.70	0.69	0.69	0.01	0.697	0.734

Abbreviation: SEM, standard error of the mean.

^a^ NC: basal diet (no supplementation); PC: NC + bacitracin methylene disalicylate (antibiotic; bacitracin: 0.03% feed); MA: NC + myristic acid; experimental unit was a pig; each least square mean represent 12 observations except for NC that represent 11 observations.

### 3.6 Correlation between mucosa‐associated microbiota and intestinal health parameters or growth performance

Helicobacteraceae was negatively correlated (*r* = −0.45; *p* < 0.05) with the ADG, whereas it was positively correlated with IgG (*r* = 0.44; *p* < 0.05), IL‐8 (*r* = 0.54; *p* < 0.05), and protein carbonyl (*r* = 0.59; *p* < 0.05; Table 11). Similarly, *Helicobacter rappini* was negatively correlated with the ADG (*r* = −0.62; *p* < 0.05) and tended to be negatively correlated with ADFI (*r* = −0.41; *p* = 0.053), whereas it was positively correlated with protein carbonyl (*r* = 0.41; *p* < 0.05) and tended to be positively correlated with IgG (*r* = 0.38; *p* = 0.078), IL‐8 (*r* = 0.40; *p* = 0.054). However, Bifidobacteriaceae was positively correlated with ADG (*r* = 0.49; *p* < 0.05) and ADFI (*r* = 0.48; *p* < 0.05) and tended to be positively correlated with villus height (*r* = 0.37; *p* = 0.074), whereas it was negatively correlated with IgG (*r* = −0.48; *p* < 0.05), IL‐8 (*r* = −0.59; *p* < 0.05), TNF‐α (*r* = −0.58; *p* < 0.05), protein carbonyl (*r* = −0.65; *p* < 0.05). At the species levels, *B. dentium* tended to be positively correlated with ADG (*r* = 0.36; *p* = 0.086) and *B. boum* was positively correlated with villus height (*r* = 0.45; *p* < 0.05), villus height to crypt depth ratio (*r* = 0.41; *p* < 0.05). Additionally, *B. boum* was negatively correlated with IL‐8 (*r* = −0.59; *p* < 0.05), TNF‐α (*r* = −0.59; *p* < 0.05), and protein carbonyl (*r* = −0.70; *p* < 0.05). Lactobacillaceae tended to be positively correlated with ADFI (*r* = 0.40; *p* = 0.053) and MDA (*r* = 0.44; *p* < 0.05), whereas it was negatively correlated with IL‐8 (*r* = −0.51; *p* < 0.05) and protein carbonyl (*r* = −0.46; *p* < 0.05). Similarly, *Lactobacillus* spp. tended to be positively correlated with ADFI (*r* = 0.36; *p* = 0.069). *Lactobacillus mucosae* was negatively correlated with IgA (*r* = −0.43; *p* < 0.05), IgG (*r* = −0.49; *p* < 0.05), IL‐8 (*r* = −0.56; *p* < 0.05), and protein carbonyl (*r* = −0.52; *p* < 0.05). *Lactobacillus equicursoris* was negatively correlated with the IgA (*r* = −0.49; *p* < 0.05) and *Lactobacillus delbrueckii* was also negatively correlated with the IL‐8 (*r* = −0.57; *p* < 0.05), TNF‐α (*r* = −0.48; *p* < 0.05) and protein carbonyl (*r* = −0.56; *p* < 0.05). Veilonellaceae and *Megasphaera* spp. were negatively correlated (*r* = −0.53; *r* = −0.59; *p* < 0.05) with IgG, respectively.

**TABLE 11:** Spearman correlation coefficients (*r*) between mucosa‐associated microbiota and other variables for intestinal health and growth performance of nursery pigs fed diets with antibiotic and myristic acid.

Item^a^	Family (*p*‐value, *r*)	Species (*p*‐value, *r*)
ADG	Helicobacteraceae (<0.05, −0.45)	*Helicobacter rappini* (<0.05, −0.62)
	Bifidobacteriaceae (<0.05, 0.49)	*Bifidobacterium thermacidophilum* (0.062, 0.39)
		*Bifidobacterium dentium* (0.086, 0.36)
		*Staphylococcus saprophyticus* (0.085, 0.36)
		*Lactobacillus salivarius* (0.092, 0.35)
ADFI	Lactobacillaceae (0.053, 0.40)	*Helicobacter rappini* (0.053, −0.40)
	Helicobacteraceae (0.093, −0.35)	*Lactobacillus mucosae* (0.091, 0.35)
	Bifidobacteriaceae (<0.05, 0.48)	*Lactobacillus* spp. (0.069, 0.38)
		*B. thermacidophilum* (0.080, 0.36)
		*S. saprophyticus* (<0.05, 0.42)
		*L. salivarius* (0.076, 0.37)
G:F	Lactobacillaceae (0.086, −0.36)	*Pseudomonas baetica fluorescens* (<0.05, 0.42)
IgA	Chlamydiaceae (<0.05, 0.49)	*L. mucosae* (<0.05, −0.43)
		*Lactobacillus delbrueckii* (0.086, −0.37)
		*Bifidobacterium boum* (0.079, −0.37)
		*Lactobacillus equicursoris* (<0.05, −0.49)
		*S. saprophyticus* (<0.05, −0.51)
		*Chlamydia muridarum* (<0.05, 0.49)
IgG	Helicobacteraceae (<0.05, 0.44)	*Helicobacter rappini* (0.078, 0.38)
	Bifidobacteriaceae (<0.05, −0.48)	*L. mucosae* (<0.05, −0.49)
	Veillonellaceae (<0.05, −0.53)	*B. boum* (0.071, −0.39)
	Pseudomonadaceae (0.081, 0.38)	*L. equicursoris* (0.072, −0.39)
	Lachnospiraceae (<0.05, −0.45)	*Helicobacter canadensis equorum* (<0.05, 0.47)
		*S. saprophyticus* (0.069, −0.40)
		*Megasphaera* spp. (<0.05, −0.59)
IL‐8	Lactobacillaceae (<0.05, −0.51)	*Helicobacter rappini* (0.054, 0.40)
	Helicobacteraceae (<0.05, 0.54)	*L. mucosae* (<0.05, −0.56)
	Bifidobacteriaceae (<0.05, −0.59)	*Lactobacillus* spp. (<0.05, −0.41)
	Erysipelotrichaceae (<0.05, −0.64)	*L. delbrueckii* (<0.05, −0.57)
	Prevotellaceae (<0.05, 0.53)	*B. boum* (<0.05, −0.59)
	Burkholderiaceae (<0.05, 0.61)	*B. thermacidophilum* (<0.05, −0.46)
		*L. equicursoris* (<0.05, −0.49)
		*H. canadensis equorum* (0.058, 0.39)
		*Megasphaera* spp. (0.060, −0.39)
		*L. salivarius* (<0.05, −0.48)
		*Ralstonia pickettii* (<0.05, 0.61)
TNF‐α	Bifidobacteriaceae (<0.05, −0.58)	*L. delbrueckii* (<0.05, −0.48)
	Pseudomonadaceae (0.087, 0.37)	*B. boum* (<0.05, −0.59)
	Erysipelotrichaceae (<0.05, −0.43)	*B. thermacidophilum* (<0.05, −0.47)
	Prevotellaceae (<0.05, 0.46)	*S. saprophyticus* (0.064, −0.40)
	Burkholderiaceae (<0.05, 0.44)	*R. pickettii* (<0.05, 0.44)
MDA	Lactobacillaceae (<0.05, 0.44)	*L. salivarius* (<0.05, 0.45)
	Ruminococcaceae (0.056, −0.41)	*R. pickettii* (<0.05, −0.46)
	Peptostreptococcaceae (<0.05, 0.48)	
	Burkholderiaceae (<0.05, −0.46)	
Protein carbonyl	Lactobacillaceae (<0.05, −0.46)	*Helicobacter rappini* (0.050, 0.41)
	Helicobacteraceae (<0.05, 0.59)	*L. mucosae* (<0.05, −0.52)
	Bifidobacteriaceae (<0.05, −0.65)	*Lactobacillus* spp. (0.086, −0.37)
	Staphylococcaceae (<0.05, −0.43)	*L. delbrueckii* (<0.05, −0.56)
	Erysipelotrichaceae (<0.05, −0.63)	*B. boum* (<0.05, −0.70)
	Prevotellaceae (<0.05, 0.69)	*B. thermacidophilum* (<0.05, −0.53)
	Ruminococcaceae (<0.05, 0.45)	*L. equicursoris* (<0.05, −0.46)
	Burkholderiaceae (<0.05, 0.55)	*H. canadensis equorum* (<0.05, 0.44)
		*S. saprophyticus* (<0.05, −0.52)
		*L. salivarius* (<0.05, −0.45)
		*R. pickettii* (<0.05, 0.55)
VH	Bifidobacteriaceae (0.074, 0.37)	*B. boum* (<0.05, 0.45)
	Pseudomonadaceae (<0.05, −0.41)	*P. baetica fluorescens* (<0.05, −0.42)
	Prevotellaceae (0.083, −0.36)	
VH:CD		*B. boum* (<0.05, 0.41)
		*P. baetica fluorescens* (0.078, −0.37)
Ki‐67^+^	Xanthomonadaceae (0.053, −0.40)	*Stenotrophomonas maltophilia* (0.060, −0.39)

Abbreviations: ADG, average daily gain; ADFI, average daily feed intake; G:F, gain to feed ratio; IgA, immunoglobulin A; IgG, immunoglobulin G; IL‐8, interleukin 8; TNF‐α, tumor necrosis factor alpha; MDA, malondialdehyde; HGMB‐1, high mobility group protein B1; VH, villus height; CD, crypt depth; VH:CD, villus height to crypt depth ratio.

## 4 DISCUSSION

Antibiotic alternatives have extensively been evaluated to reduce intestinal challenges from weaning stress and improve the intestinal health and growth of newly weaned pigs without antibiotics. Some successful alternatives include, but are not limited to, organic acids (Choi et al., 2023; Radcliffe et al., 1998), phytobiotics (Duarte & Kim, 2022b; Wang et al., 2022), prebiotics (Flickinger et al., 2003), probiotics (Roselli et al., 2017; Sun et al., 2021), postbiotics (Cheng et al., 2021; Mathew et al., 1998; Xu et al., 2022), antimicrobial peptides (Poudel et al., 2022; Roh et al., 2015), fatty acids (Gebhardt et al., 2020; Moita et al., 2021), feed enzymes (Chen et al., 2020; Moita et al., 2022; Petry & Patience, 2020), and chelated minerals (Coffey et al., 1994; Jang et al., 2023) with antimicrobial properties. Among these alternatives, myristic acid has received increased attention for its antimicrobial properties (Boyen et al., 2008; Liu, 2015), which can positively modulate jejunal mucosa‐associated microbiota, decrease intestinal inflammation, and improve the growth performance of nursery pigs.

Myristic acid, a saturated fatty acid consisting of a chain of 14 carbon atoms with a carboxyl group, has been shown to have antimicrobial properties, especially against Gram‐positive pathogenic bacteria (Qi et al., 2023). Myristic acid can attach to the cell walls of opportunistic pathogenic bacteria, altering bacterial cell membrane permeability and exhibiting antimicrobial activity (Chen et al., 2019; Liu & Huang, 2012).

These antimicrobial properties of myristic acid can positively modulate the microbiota in the jejunal mucosa of pigs in this study. The increased Firmicutes in the jejunal mucosa due to myristic acid was associated with the increased Lactobacillaceae and Veilollaceae in this study. Myristic acid has demonstrated high antimicrobial activity against various opportunistic Gram‐positive bacteria, including *Clostrium perfringens* (Qi et al., 2023), *S. aureus* (Park et al., 2022), and *Streptococcus agalactiae* (Kelsey et al., 2006), indicating that myristic acid may reduce opportunistic pathogenic Gram‐positive bacteria and increase beneficial bacteria in the jejunum of pigs. One possible reason for its antimicrobial activity against the opportunistic pathogenic Gram‐positive bacteria is likely that these bacteria have highly D‐alanylated lipoteichoic acids in the cell walls, which increase the positive charge of the cell walls (Neuhaus & Baddiley, 2003), potentially allowing relatively higher interaction with fatty acids (Shin et al., 2024). Another possible reason for the reduction of opportunistic pathogenic Gram‐positive bacteria with myristic acid may be the absence of lipopolysaccharides, which are present in Gram‐negative bacteria (Hancock, 1997). The O‐antigen, located in the outermost portion of the lipopolysaccharides in Gram‐negative bacteria, makes it difficult for hydrophobic molecules, such as fatty acids, to attach the cell wall, disrupt bacterial structure, and interfere with bacterial enzymes required for growth (Desbois & Smith, 2010; Silhavy et al., 2010). Supplementation with antimicrobial compounds also reduced pathogenic bacteria and increased the beneficial bacteria in the enterocytes due to reduced colonization of ammonia‐producing bacteria (Choi & Kim, 2024; Duarte & Kim, 2022a), which may explain myristic acid increased Firmicutes and decreased Proteobacteria in this study.

Having more direct interactions with the mucus layer and intestinal cells (Arpaia et al., 2013; Duarte et al., 2023; Duarte & Kim, 2022c), jejunal mucosa‐associated microbiota plays an important role in the intestinal health of pigs, as it is closely related to the immune system, nutrient metabolism, and resistance to pathogenic colonization compared with luminal microbiota (Kim & Duarte, 2021) The improved bacterial diversity parameters, as indicated by Chao 1, Shannon, and Simpson indexes, along with the changes in Bray‐Curtis distance in beta diversity, enhanced the intestinal health of nursery pigs (Ramayo‐Caldas et al., 2016), indicating myristic acid and bacitracin positively impact intestinal health of pigs. In this study, supplementation of myristic acid in feeds increased the RA of Bifidobacteriaceae and Veilonellaceae in the jejunal mucosa of nursery pigs, both of which belong to Actinobacteria and Firmicutes, respectively. Coconut oil supplementation, containing also 16 to 21% myristic acid (Nitbani et al., 2022), increased the *Bifidobacterium* in the cecal microbiota (Rolinec et al., 2020) and decreased *E. coli* population in the digesta and mucosa in the ileum of pigs (López‐Colom et al., 2019). Additionally, replacing soybean oil with black soldier fly larvae oil in feeds, containing 6 to 25% myristic acid (Kim et al., 2021), increased Veilonellaceae in the cecal microbiota of broilers (Chen et al., 2022) and replacing corn oil with black soldier fly larvae oil improved growth performance in chickens (Kim et al., 2020) and nursery pigs (van Heugten et al., 2022). These positive modulations to intestinal microbiota from coconut oil or black soldier fly larvae oil may be due to antimicrobial effects of myristic acid on the intestinal microbiota and intestinal health of pigs. However, coconut oil and black soldier fly larvae oil also contained 45 to 52% and 27 to 41% lauric acid content, respectively (Nitbani et al., 2022), which may cause confounding effects on the intestinal microbiota and intestinal health of pigs, as lauric acid supplementation in feeds also improved intestinal health of nursery pigs (Ficagna et al., 2024; Zeng et al., 2022).

*Bifidobacterium* is known for degrading polysaccharides in feedstuffs, producing short‐chain fatty acids (SCFA) in the intestinal tract of pigs (Wu et al., 2011). The Bifidobacteriaceae and Veillonellaceae in the jejunal mucosa of nursery pigs strongly also correlated with improved intestinal health parameters including TNF‐α, protein carbonyl, and VH (Duarte & Kim, 2022c), supported the correlation association results of this study. Immune cells like dendritic cells and microfold cells in the jejunum detect opportunistic pathogenic bacteria via toll‐like receptors and other sensors, which help regulate cytokine production to enhance the immune system, intestinal health, and growth of pigs (Qi et al., 2017). Myristic acid supplementation reduced levels of Proteobacteria, which included *Helicobacter rappini*, negatively correlating with the growth performance of pigs in this study. The *Helicobacter rappini* in the jejunal mucosa was negatively correlated with the VH:CD of nursery pigs (Duarte & Kim, 2022c). Additionally, *Megasphaera* and *Ruminococcus* are known for increasing butyrate production in the intestine of pigs (Claesson et al., 2012; Duncan et al., 2004; Hashizume et al., 2003), as well as for reducing diarrhea incidence and intestinal permeability of small intestine (Zhang, Zhang, et al., 2022). Additionally, high SCFA contents in the digesta positively influenced the intestinal microbiota of pigs (Qi et al., 2021). These positive modulations of microbiota by myristic acid may have contributed to reduced levels of IL‐8 and IgG in the jejunal mucosa of nursery pigs in this study. Myristic acid directly activated the GPR84 located in the jejunum (Wang et al., 2006), and activation of GPR84 can enhance the pro‐inflammatory cytokines (Zhang, Chen, et al., 2022), which is another possible reason why myristic acid reduced the immune responses in this study. The reduction in IL‐8 can reduce the release of macrophages producing pro‐inflammatory cytokines, thus reducing inflammation and excessive energy expenditure in the intestine of pigs (Huang & Lee, 2018). Interestingly, myristic acid supplementation in this study did not decrease IgA content in jejunal mucosa, whereas it decreased IgG content. This discrepancy may be attributed to the longer response time of IgG compared with IgA after weaning stress in the intestine of nursery pigs. Another potential possibility for the improved immune responses in this study may be due to the contribution of myristic acid as an energy source for the intestinal health of pigs (Moita et al., 2021; Rosero et al., 2015). In this study, myristic acid supplementation, however, did not increase the AID of energy and nutrients. The reason for this may be due to not having enough fatty acid contents to increase the AID of energy and nutrients. Overall, myristic acid supplementation has a more pronounced impact on immune responses, influencing intestinal health rather than improving nutrient utilization in nursery pigs, and thus positively contributing to the enhancement of growth performance of nursery pigs.

The bacitracin supplementation in feeds increased the RA of *Lactobacillus* and *Mitsuokella* and also improved the Simpson index and was negatively correlated with increased immune responses and oxidative damage products and positively correlated with growth performance in this study. Previous studies also reported that the positive modulation of mucosa‐associated microbiota by antibiotics reduced inflammatory responses, enhanced intestinal morphology, and increased the growth performance of nursery pigs (Duarte & Kim, 2022a; Sun et al., 2021; Xu et al., 2022). However, in comparison with the effects of bacitracin on the growth performance of pigs in this study, the effects of myristic acid require a longer period. The reason for this might be due to the adaptation period needed to modulate mucosa‐associated microbiota by dietary intervention in the intestinal tract of pigs (Castillo et al., 2007; Le Sciellour et al., 2018). The NSP‐degrading and SCFA‐producing bacteria, such as Bifidobacteriaceae and Ruminococcaceae, increased as the adaptation period extended from d 7 to 21 (Gao et al., 2023), indicating modulation of mucosa‐associated microbiota requires time. Consequently, the increased SCFA‐producing bacteria including *Bifidobacterium* and Ruminococcaceae can improve the intestinal health and growth performance of pigs. In previous studies, dietary SCFA supplementation, however, did not improve the growth performance of nursery pigs (Fang et al., 2014; Hou et al., 2006; Valencia & Chavez, 2002). Furthermore, the 0.1% sodium acetate supplementation increased the release of peptide YY and glucagon‐like peptide‐1 in serum, while reducing the feed intake of pigs (Jiao et al., 2020). The deviation between this study and the previous studies could be attributed to the variations in the types and contents of fatty acids present in feeds, which potentially influence the growth performance of nursery pigs.

In conclusion, both bacitracin and myristic supplementation improved the intestinal microbiota, intestinal health, and growth performance of nursery pigs. However, in bacterial diversity indexes, the myristic acid improved Chao 1, Shannon, and Simpson indexes, whereas bacitracin only improved the Simpson index compared with the basal diet. Moreover, myristic acid increased RA of *Bifidobacterium* and *Megasphaera* in the jejunal mucosa, whereas bacitracin increased *Lactobacillus* and *Mitsuokella* in the mucosa. The effects of bacitracin on intestinal health and growth performance were rather immediate, whereas the effects of myristic acid were obtained after a 3‐week feeding. The strong correlation between Lactobacillaceae or Bifidobacteriaceae and the intestinal health and growth performance parameter in nursery pigs indicates that beneficial microbiota highly influence intestinal health and growth.

## CONFLICT OF INTEREST STATEMENT

There is no conflict of interest regarding the material discussed in this study.
